# The diagnostic and prognostic value of CXCL13, CXCL10, and CXCL8 in patients with neurosyphilis

**DOI:** 10.3389/fimmu.2025.1654251

**Published:** 2025-10-27

**Authors:** Hongjing Guan, Jingli Peng, Zihao Xia, Xiaoyun Di, Qin Wang, Chunmiao Zou, Rentian Cai, Chen Chen, Hongxia Wei

**Affiliations:** ^1^ Department of Infectious Diseases, The School of Public Health of Nanjing Medical University, The Second Hospital of Nanjing, Jiangsu, Nanjing, China; ^2^ Department of Infectious Diseases, The Second Hospital of Nanjing, Affiliated to Nanjing University of Chinese Medicine, Jiangsu, Nanjing, China

**Keywords:** neurosyphilis, CXC chemokine ligand 13, HIV, diagnosis, model construction

## Abstract

**Background:**

The purpose of this study is to examine the diagnostic and therapeutic value of CXCL13 (CSF-CXCL13), CXCL10 (CSF-CXCL10), and CXCL8 (CSF-CXCXCL8) in NS patients in a systematic manner.

**Method:**

The study will include individuals who are the first to undergo neurosyphilis (NS) screening from August 2023 to October 2024, and will gather demographic, clinical, and laboratory data, as well as cerebrospinal fluid (CSF) and blood samples. Enzyme-linked immunosorbent assay (ELISA) was used to quantitatively detect the concentrations of CXCL13, CXCL10, and CXCL8 in CSF and blood samples. Use receiver operating characteristic (ROC) curves to evaluate the ability of cytokines to distinguish between NS and non-NS individuals, and further evaluate in different populations, including the total population, People Living with HIV(PLWH), Non-People Living with HIV(Non-PLWH) population. Develop an NS diagnostic model using logistic regression analysis results, and ensure the model is valid by conducting 5-fold cross-validation, calibration curve, and clinical decision curve (DCA). Use a Nomogram to visualize the model.

**Result:**

A total of 233 participants were included in the study. ROC shows that the area under the curve (AUC) of CSF-CXCL13 in distinguishing NS from Non-NS,#x3001; NS from CNS infections is 0.812 and 0.839, respectively. In contrast, the AUC of CSF-CXCL10 and CSF-CXCL8 in distinguishing NS from Non-NS were 0.568 and 0.638, respectively. The AUC in distinguishing NS from other CNS infections were 0.604 and 0.556, respectively. To enhance the effectiveness of differential diagnosis, we employed logistic regression analysis to screen variables and developed a predictive model MODEL1. The results showed that the AUC value of MODEL1 was 0.888, and the calibration curve and DCA curve demonstrated good accuracy and clinical benefits of the model, demonstrating good predictive performance. After NS treatment, the levels of CSF-CXCL13, CSF-CXCL10, and CSF-CXCL8 slightly decreased.

**Conclusion:**

CSF-CXCL13 has good differential value in distinguishing NS from Non-NS, NS from CNS infections, while CSF-CXCL10 and CSF-CXCL8 have lower differential sensitivity. The diagnostic performance of the NS diagnostic model (Model 1) based on CSF-CXCL13 has been improved.

## 1 Introduction

Syphilis is a sexually transmitted disease caused by infection with Treponema pallidum. Neurosyphilis (NS) can be caused by Treponema pallidum if it penetrates the blood-brain barrier and invades the central nervous system. At present, Treponema pallidum particle agglutination assay(TPPA), toluidine red unheated serum test(TRUST), venereal disease research laboratory(VDRL), fluorescent treponemal antibody-absorption(FTA-ABS) and other methods for diagnosing NS may have an imbalance in sensitivity and specificity, so it is not recommended to use them in clinical practice (1–3).

Previous studies have shown that C-X-C motif chemokine 13(CXCL13), CXC chemokine ligand 10(CXCL10), and C-X-C motif chemokine ligand 8(CXCL8) in cerebrospinal fluid(CSF) are abnormally elevated in patients with other CNS infections, such as brucellosis, multiple sclerosis, and facial nerve paralysis (4–6). CSF-CXCL13 concentration increased significantly in NS patients, which was higher than in non-NS patients (5). According to Ling Yang et al.’s study, CXCL13 levels in NS patients may be 40.8 times higher than those in non-NS syphilis patients (7). It was found that there was no significant difference in sensitivity and specificity between CSF-CXCL13 and VDRL when compared to diagnosing NS. In Asymptomatic neurosyphilis(ANS) patients, the diagnostic performance of CSF-CXCL13 is even better than VDRL (8). CSF-CXCL13 has high diagnostic sensitivity and specificity, and is expected to become an auxiliary diagnostic tool for NS (5, 7).

However, previous studies have not confirmed the ability of CSF-CXCL13, CSF-CXCL10, and CSF-CXCL8 to distinguish NS from other CNS (such as tuberculous meningitis and cryptococcal meningitis), and some research samples are too small (only a dozen cases), resulting in some bias in the results (8). Overall, in previous studies, the detection samples for CSF-CXCL13, CSF-CXCL10, and CSF-CXCL8 were too small, NS was defined only by Rapid Plasma Reagin Card Test(RPR) or VDRL in CSF, which was not comprehensive and unclear about its prognostic value for NS patients. Therefore, this study aims to evaluate the value and therapeutic potential of CXCL13, CXCL10, and CXCL8 in CSF as auxiliary diagnostic indicators for NS through a larger research sample and a more rigorous definition of NS.

## 2 Methods

### 2.1 Research object

This study is a prospective study that collected CSF samples from patients who underwent lumbar puncture for the first time to screen for neurosyphilis between August 2023 and October 2024, as well as CSF samples during follow-up NS. Demographic, clinical, and laboratory data were gathered, including those who are living with HIV (PLWH) and those who are not.

Inclusion criteria:

1. Adult patients aged ≥ 18 years, regardless of gender.
2. Blood syphilis antibody positive.
3. Initial lumbar puncture screening for NS, accompanied by complete CSF routine, biochemical, and syphilis antibody test results.
4. Other CNS infections, such as cryptococcal meningitis, varicella encephalitis, Cytomegalovirus encephalitis, Tuberculous meningitis, herpes zoster neuralgia and CNS infectious(Mixed infections of several types).

Exclusion criteria:

1. Key data missing (CSF-TRUST or CSF-TPPA or CSF-WBC or CSF-PRO missing).

This study has been approved by the Medical Ethics Committee of Nanjing Second Hospital and meets the requirements of medical ethics (ethics approval number 2023-LS-ky-033).

### 2.2 Research methods

This study is a prospective study, dividing the research subjects into three groups: syphilis(Non-NS), NS, and other CNS infections(No syphilis infection).

#### 2.2.1 Data collection

While collecting CSF and blood samples from patients, we also collected demographic and clinical data through the hospital’s LIS and HIS systems, including age, gender, marital status, HIV status, peripheral blood indicators (absolute neutrophil count, neutrophil percentage, absolute eosinophil count, eosinophil percentage, absolute eosinophil count, eosinophil percentage, Toluene Red Untreated Serum Test Titer in Blood(sero-TRUST), Treponema pallidum particle agglutination test titer in Blood(sero-TPPA), Routine biochemical indicators of CSF include white blood cells in cerebrospinal fluid (CSF-WBC), proteins in cerebrospinal fluid (CSF-PRO), TRUST titers in cerebrospinal fluid (CSF-TRUST), and TPPA in cerebrospinal fluid (CSF-TPPA), clinical symptoms, syphilis treatment, HIV treatment, and magnetic resonance imaging (MRI) results. Detect the collected CSF and blood samples using the CXCL13(Human CXCL13/BCA-1/BLC ELISA Kit; elabscience)、 The human interferon inducible protein 10 (IP-10/CXCL10) enzyme-linked immunosorbent assay kit (Human CXCL10 ELISA KIT; Wuhan Huamei) and human interleukin-8 (IL-8; CXCL8) enzyme-linked immunosorbent assay kit (Human Interleukin-8, IL-8 ELISA KIT; Wuhan Huamei) were used for detection.

#### 2.2.2 Sample size calculation

Using PASS software for sample size calculation, considering that NS, non NS, and other central nervous system infection groups will be collected in the study, only ROC curves will be compared between NS and other central nervous system infection groups in the study design, and sample size calculation will be carried out separately. We used a case-control study to calculate the sample size of NS and non NS groups, and selected “Tests for Two Related Proportions in a Matched Case Control Design”. The parameter settings are as follows: POWER:0.8、Alpha:0.15、 According to 1:1 matching, the OR value is: 3. The expected incidence rate is 20%, and the lost follow-up rate is 20%. The final calculation result shows that 69 cases are included in the case group and the control group respectively.

We used the ROC method to calculate the sample size for NS and other central nervous system infections. We selected “ROC” and set the parameters as follows: AUC 0 is 0.8, AUC 1 is 0.90, and the deletion rate is 20%. Finally, 65 cases of other central nervous system infections should be included.

#### 2.2.3 Experimental procedure

##### 2.2.3.1 Sample and equipment preparation:

Follow the instructions of the ELISA kit for operation.

##### 2.2.3.1 Repetitive testing:

During the sample testing process, we randomly select a few samples from each batch for repeatability testing to maintain the applicability of the results. We use OD mean, OD standard deviation, and OD coefficient of variation to reflect the differences in test results between different batches, mainly through the coefficient of variation (see **Supplementary Table 1**).

#### 2.2.4 Model validation program

##### 2.2.4.1 5-Fold cross-validation

Divide the training dataset evenly into 5 subsets (each referred to as a “fold”), using 4 subsets as the training set and the remaining 1 subset as the testing set each time. Model training and evaluation: Train the model using the current training set and calculate performance metrics (such as Accuracy, ROC, F1-Score, Precision.) on the corresponding test set. Repeating iterations, changing different test sets each time (a total of 5 combinations), with different training and testing samples each time, ultimately resulting in 5 performance indicators. We constructed a dataset of 190 samples for our model, so we chose 5-fold cross-validation.

##### 2.2.4.2 Bootstrap

During the calibration curve drawing process, Bootstrap method is used, which is a non-parametric approach to estimate statistical distribution by repeatedly sampling from a dataset. It can be used to construct hypothesis testing. When there is doubt about the assumptions of the parametric model, or when statistical inference based on the parametric model is not feasible and complex formulas are required to calculate standard error, self-help methods can be used as an alternative. The requirement for resampling samples is the same as the original sample size (N), with 1 sample extracted from the dataset each time there is a replacement, and repeated N times to form a new dataset. We can have a simple understanding of Bootstrap. The new dataset is an approximate distribution of the research sample, which in turn approximates the population distribution. Therefore, the resampled data distribution approximates the population distribution. Use resampled samples to calculate probabilities and generate predicted probabilities for corresponding observations. Compare the predicted probability with the actual probability to form a scatter plot, thereby creating a calibration curve. Simultaneously use optimm-corrected estimates to correct the results, including C-index Brier, Intercept, Slope.

### 2.3 Research definition

#### 2.3.1 NS definition

According to the Diagnosis and Treatment Guidelines for Syphilis, Gonorrhea, and Chlamydia trachomatis Infection in the Reproductive Tract (2020), The definition of NS must meet the following conditions:

##### 2.3.1.1 Clear diagnosis of NS

1. Patients with NS related epidemiological history, sero-TRUST positive and sero-TPPA positive, CSF-TRUST positive and CSF-TPPA positive, and/or CSF-PRO ≥ 500g/L and/or CSF-WBC ≥ 5 × 106/L, with or without NS related clinical symptoms.

##### 2.3.1.2 Suspected diagnosis of NS

Patients with a history of NS related epidemiology, sero-TRUST positive and sero-TPPA positive, CSF-TRUST negative and CSF-TPPA positive, CSF-PRO<500g/L and CSF-WBC<5 × 106/L, with or without NS related clinical symptoms.

#### 2.3.2 Syphilis

Patients with positive syphilis antibodies are excluded from NS.

#### 2.3.3 CNS infections

Including cryptococcal meningitis, varicella encephalitis, cytomegalovirus encephalitis, tuberculous meningitis, herpes zoster neuralgia, and CNS infections (Mixed infections of several types).

### 2.4 Statistical analysis

The type of data determines which data analysis method is chosen. If the continuous variable is normally distributed, it is represented by its mean + standard deviation. Otherwise, the median (interquartile range, IQR) is used to represent it. Inter group comparisons are conducted using parametric tests (t-test, analysis of variance) and nonparametric tests (Wilcoxon rank sum test); Categorical variables are described using frequency and percentage, and comparison between groups is performed using X2 test or Fisher’s exact probability method. Use Excel to construct a logarithmic standard curve based on the sample’s OD value, and use the standard curve to convert the absorbance value to a specific concentration. During the detection process of different cytokines, some samples will be randomly selected for repetitive testing. The results of the repetitive testing are shown in the **Supplementary Table 1**. Wilcoxon rank sum test was used to compare the differences in cerebrospinal fluid CXCL13 (CSF-CXCL13), CSF-CXCL10 (CSF-CXCL10), and cerebrospinal fluid CXCL8 (CSF-CXCL8) between syphilis, NS, and CNS infection groups. Spearman analysis was performed to determine the relationship between CSF-CXCL13, CSF-CXCL10, CSF-CXCL8, and inflammatory factors in peripheral blood, and the correlation between each variable was visualized using a heatmap. The ROC curve reflects the ability of CSF-CXCL13, CSF-CXCL10, and CSF-CXCL8 to distinguish between NS and Non-NS, and is analyzed separately in PLWH and non-PLWH. The difference between AUC is compared using the Delong test. Correction of P-values using Bonferroni test in multi-subgroup comparisons. To avoid the bias caused by excessive ROC, we also introduce metrics such as Recall, Precision, F1-Score, Accuracy to evaluate the results. In order to further improve diagnostic efficiency, logistic univariate and multiple regression were used to screen model variables, and Model 1 was constructed based on this to diagnose NS. Considering the research sample and the lack of external validation, we conducted a 5-fold cross-validation within the data to ensure the stability of the results. The calibration curve and Clinical Decision Curve (DCA) were used to evaluate the model’s effectiveness and clinical usability. Use Mann Kendall trend test and time series analysis to examine the changes in cytokines and sero-TRUST after treatment.

### 2.5 Research flowchart

The research flowchart is shown in **Supplementary Figure 1**.

## 3 Results

### 3.1 Differences in levels of CSF-CXCL13, CSF-CSF-CXCL10, and CSF-CXCL8 between NS and non-NS patients

**Figure 1:**
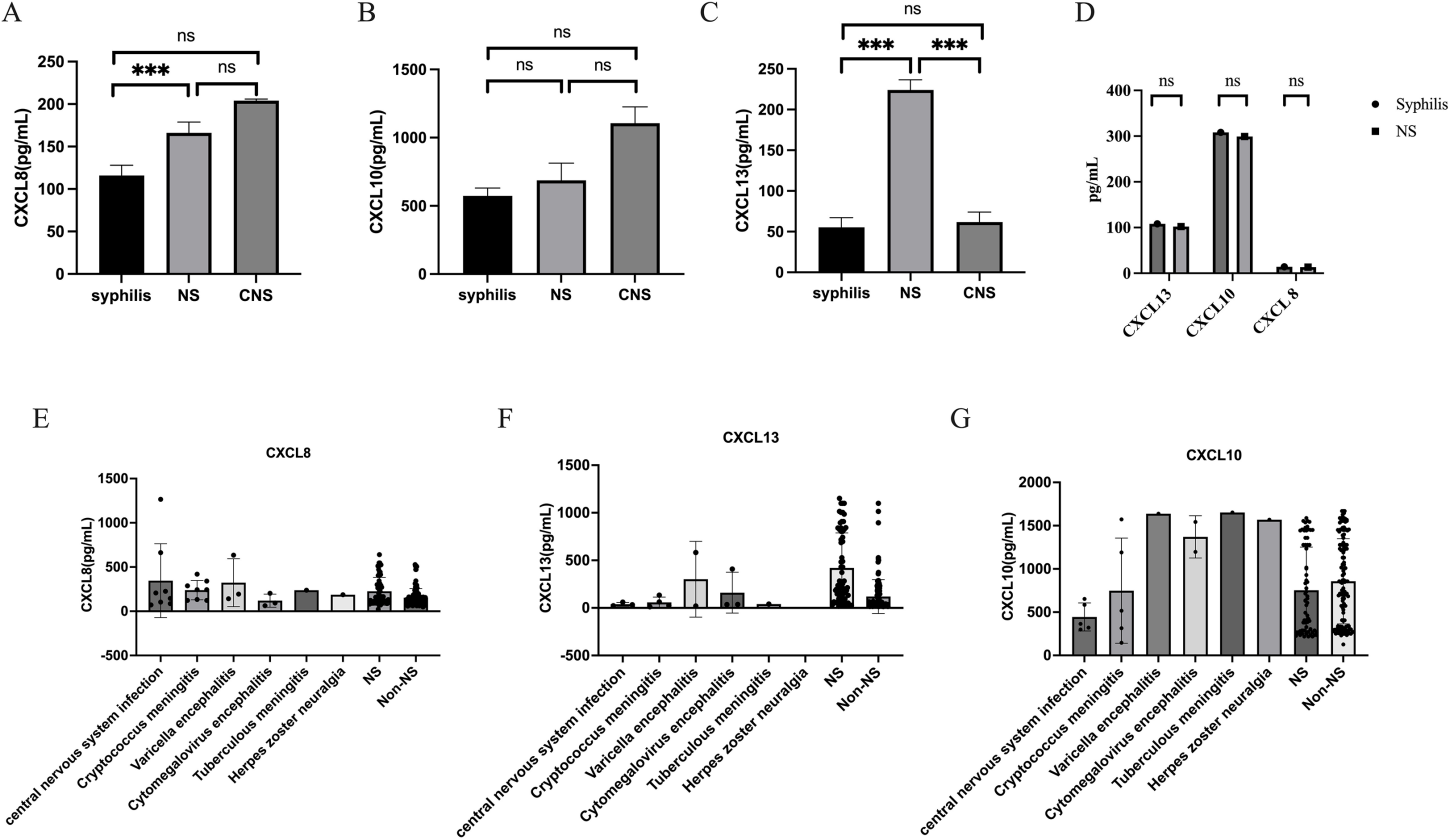
CSF-CXCL13, CSF-CXCL10, and CSF-CXCL8 are at different levels among the three population groups. **(A)** Levels of CXCL13 in cerebrospinal fluid of syphilis ,neurosyphilis ,other central nervous system populations. **(B)** Levels of CXCL10 in cerebrospinal fluid of syphilis , neurosyphilis ,other central nervous system populations. **(C)** Levels of CXCL8 in cerebrospinal fluid of syphilis ,neurosyphilis ,other central nervous system populations. **(D)** The levels of CXCL13, CXCL10, and CXCL8 in the blood of NS and Non NS. **(E)** CXCL13 levels in different types of CNS infections and non-NS infections. **(F)** CXCL10 levels in different types of CNS infections and non-NS infections **(G)** CXCL8 levels in different types of CNS infections and non-NS infections. *** represents statistical differences in the results.

This study is a prospective study that collected CSF samples from patients who underwent lumbar puncture screening for neurosyphilis for the first time between August 2023 and October 2024, as well as CSF samples from patients who received NS treatment during follow-up. 233 CSF samples were taken from 66 NS patients, 124 syphilis patients, and 43 patients with CNS infections in this study. It’s worth noting that syphilis was not present in any of the patients with CNS infection. Prospective collection of 36 blood samples resulted in 22 cases of syphilis and 14 cases of NS. Calculate specific concentrations of CXCL13, CXCL10, and CXCL8 based on standard curves (**Supplementary Figure 2**). The inter batch variations of different ELISA reagents are shown in **Supplementary Table 1**. The research results showed that the levels of CXCL13 in the CSF of NS patients were significantly higher than those of syphilis patients and other CNS infection patients, while there was no statistically significant difference in CXCL13 levels between syphilis patients and other CNS infection patients (**Figure 1A**). The levels of CXCL10 in CSF were not significantly different among the three groups of patients (**Figure 1B**). In addition, patients with NS had a higher CXCL8 level in their CSF compared to patients with syphilis and other CNS infections. Even though the levels of CXCL8 in the CSF of other CNS infection patients were greater than those of syphilis patients, the difference was not significant (**Figure 1C**). **Figure 1D** displays the blood levels of CXCL13, CXCL10, and CXCL8 of the patient. The levels of CXCL13, CXCL10, and CXCL8 in different types of CNS infections are shown in **Figures 1E–G**.

**Table 1** provides detailed information on the specific baseline characteristics of the population.

**Table 1:** Prospective population baseline characteristics.

Variables	Non-NS (N=124)	NS (N=66)	p.values
Whethertherearesymptomsornot (n,%)			<0.001
NO	111 (89.5%)	30 (45.5%)	
YES	13 (10.5%)	36 (54.5%)	
syphilistreated (n,%)			0.186
NO	67 (54.0%)	43 (65.2%)	
YES	57 (46.0%)	23 (34.8%)	
CXCL13	37.1 [22.1;178]	246 [136;816]	<0.001
CXCL10	801 [333;1284]	634 [283;1277]	0.121
CXCL8	123 [91.2;170]	157 [111;310]	0.002
Age	37.0 [30.8;52.0]	42.0 [29.5;57.8]	0.282
Sex (n,%)			0.824
female	14 (11.3%)	6 (9.09%)	
male	110 (88.7%)	60 (90.9%)	
sero-TRUST			0.023
≤1:16	86 (69.4%)	34 (51.5%)	
>1:16	38 (30.6%)	32 (48.5%)	
Chlorine	126 [123;127]	125 [122;128]	0.966
CSF-PRO	234 [163;322]	450 [272;600]	<0.001
Glucose	3.60 [3.30;3.90]	3.50 [3.12;4.00]	0.894
Lactate dehydrogenas	17.0 [12.0;21.0]	18.0 [13.2;23.0]	0.219
Adenosine deaminase	0.00 [0.00;2.00]	0.00 [0.00;3.00]	0.657
Is there a cavity obstruction			1.000
NO	115 (92.7%)	61 (92.4%)	
YES	9 (7.26%)	5 (7.58%)	

### 3.2 Diagnostic efficacy of CSF-CXCL13, CSF-CXCL10 and CSF-CXCL8

**Table 2** demonstrates that CXCL13 in CSF is the most effective diagnostic tool for distinguishing NS from non-NS. The AUC is 0812 with a sensitivity of 0.909 and a specificity of 0.556. Both CXCL10 and CXCL8 had low diagnostic sensitivity and poor diagnostic performance, with the AUC values of 0.568 and 0.638 in CSF respectively, indicating low diagnostic sensitivity and specificity. Furthermore, there is a substantial difference in AUC values among the three.

**Table 2:** Performance of ROC curves in different subgroups.

Subgroup	Model	P.values	Bonferroni	AUC	95%CI	Specificity	Sensitivity	Accuracy	Precision	Recall
Total	CXCL13	<0.001	<0.001	0.812	0.752-0.872	0.556	0.909	0.679	0.522	0.909
	CXCL10	0.940	1.000	0.568	0.481-0.656	0.831	0.303	0.647	0.488	0.303
	CXCL8	0.001	0.003	0.638	0.553-0.722	0.839	0.409	0.689	0.574	0.409
PLWH	CXCL13	<0.001	<0.001	0.770	0.680-0.860	0.971	0.459	0.835	0.850	0.459
	CXCL10	0.934	1.000	0.584	0.475-0.693	0.598	0.622	0.604	0.359	0.622
	CXCL8	0.004	0.012	0.648	0.533-0.764	0.853	0.514	0.763	0.559	0.514
Non-PLWH	CXCL13	<0.001	<0.001	0.852	0.753-0.952	0.741	0.903	0.828	0.800	0.903
	CXCL10	0.680	1.000	0.535	0.382-0.688	0.963	0.290	0.603	0.900	0.290
	CXCL8	0.029	0.086	0.646	0.503-0.790	0.407	0.903	0.672	0.636	0.903
Clarify diagnostic	CXCL13	<0.001	<0.001	0.943	0.882-1.000	0.948	0.850	0.923	0.850	0.850
	CXCL10	0.151	0.454	0.578	0.431-0.725	0.690	0.550	0.654	0.379	0.550
	CXCL8	<0.001	<0.001	0.767	0.634-0.901	0.897	0.600	0.821	0.667	0.600
Suspected diagnostic	CXCL13	<0.001	<0.001	0.740	0.652-0.829	0.542	0.894	0.681	0.560	0.894
	CXCL10	0.998	1.000	0.655	0.551-0.759	0.653	0.660	0.655	0.554	0.660
	CXCL8	0.050	0.149	0.590	0.485-0.695	0.389	0.787	0.546	0.457	0.787
NS and CNS	CXCL13	<0.001	<0.001	0.839	0.729-0.949	0.813	0.742	0.756	0.942	0.742
	CXCL10	0.902	1.000	0.604	0.438-0.771	0.313	0.939	0.817	0.849	0.939
	CXCL8	0.799	1.000	0.556	0.423-0.689	0.615	0.591	0.598	0.796	0.591
Model 1		<0.001	/	0.888	0.840-0.936	0.815	0.864	0.832	0.713	0.864
CXCL13+CXCL10+CXCL8		<0.001	/	0.808	0.747-0.869	0.847	0.621	0.768	0.683	0.621

### 3.3 Diagnostic efficacy of CSF-CXCL10 and CSF-CXCL8 in PLWH, non-HIV, suspected NS, and accurately diagnosed NS populations

We observed the effectiveness of CXCL13, CXCL10, and CXCL8 in distinguishing NS from Non NS in the general population, HIV positive population, and HIV negative population (ROC curves). In populations with only NS and other CNS infections, we will examine the ability of CXCL13, CXCL10, and CXCL8 to differentiate NS from other CNS infections. **Table 2** indicates that CXCL13 in CSF has a high diagnostic specificity in PLWH, but it also has a relatively low sensitivity, with an AUC of 0.770. In contrast, the AUC values of CSF-CXCL10 and CSF-CXCL8 are lower, at 0.584 (sensitivity: 0.622, specificity: 0.598) and 0.648 (sensitivity: 0.514, specificity: 0.853), respectively. The highest AUC value of CSF-CXCL13 is found in non-PLWH infective populations (**Table 2**), which indicates high diagnostic sensitivity with an AUC of 0.852 (sensitivity: 0.903, specificity: 0.741). The AUC of CSF-CXCL10 and CSF-CXCL8 was 0.535 (sensitivity: 0.290, specificity: 0.963) and 0.646 (sensitivity: 0.903, specificity: 0.407), respectively. In addition, CSF-CXCL13 exhibits high sensitivity in distinguishing CNS infections from NS, with an AUC of 0.839 (sensitivity: 0.813, specificity: 0.742), while CSF-CXCL10 and CSF-CXCL8 had lower sensitivity, with AUC of 0.604 (sensitivity: 0.939, specificity: 0.313) and 0.556 (sensitivity: 0.591, specificity: 0.615), respectively (**Table 2**). The diagnostic ability is still good in NS patients with suspected and confirmed diagnoses. The sensitivity, specificity, and accuracy of ROC curves in different subgroups are shown in **Table 2**.

### 3.4 Constructing a diagnostic model based on variables such as CSF-CXCL13

**Figure 2:**
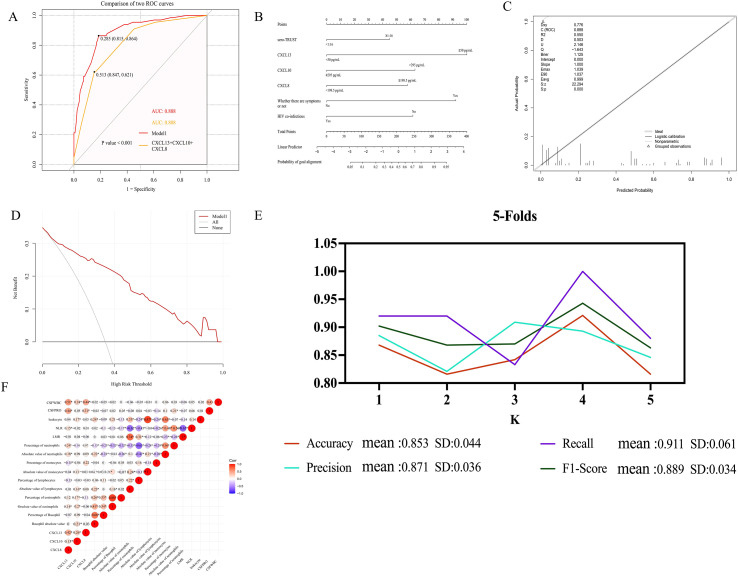
The ROC curve and column chart of Model 1. **(A)** ROC curve based on logistic modeling. Note: MODEL 1 is based on logistic regression to construct a model, and CSF-CXCL13+CSF-CXCL10+CSF-CXCL8 is a combined diagnosis of three cytokines in CSF. **(B)** Column chart of model MODEL1. **(C)** Calibration curve of Model 1. **(D)** DCA curve of Model **(E)** Changes in Accuracy, Precision, Recall, F1 Score under 5-fold cross-validation **(F)** The correlation between cytokines such as CSF-CXCL13 and inflammatory markers in peripheral blood. The “*” in the heatmap represent statistical differences.

To identify factors that are associated with the occurrence of NS, include variables with a P value of **<**0.1 in logistic univariate regression in multivariate analysis. The multivariate results in **Table 3** showed that sero-TRUST ≥ 1:16 (OR (95CI%): 2.47 (1.06-5.74), symptomatic (OR (95CI%): 7.33 (3.04-17.67), P<0.001), CSF-CXCL13 ≥ 50pg/mL (OR (95CI%): 5.38 (2.03-14.25, P<0.001)), CSF-CXCL8 ≥ 198.5pg/mL (OR (95CI%): 3.29 (1.23-8.79), P<0.001) increased the risk of developing NS, while HIV (OR (95CI%): 0.22 (0.09-0.54), P<0.001) decreased the risk of developing NS. **Supplementary Table 2** shows the VIF values of multiple variables. Subsequently, we incorporated the factors from the multiple factor analysis into Model 1,The specific diagnostic capability of the model is shown in **Figure 2A**. We chose 5-fold cross-validation because of the study**’**s large sample size. The model**’**s 5-fold cross-validation results confirmed that accuracy, recall, precision, and F1 score remained constant and above 0.8 (**Figure 2E**). **Figure 2B** displays the nomogram. **Figure 2C** shows the calibration curve, and the results are corrected using bootstrap (Bootstrap=500) and optimistic corrected. The corrected C-index, Brier, Intercept, and Slope results are shown in **Table 4**. It was found that the model had good accuracy. Within the threshold probability range of 0.05-0.98, the clinical net benefit was guided by the intervention based on the predicted probability of Model 1 diagnosis of NS was higher than that of all individuals**’** intervention (all) and all individuals**’** no intervention (none) (**Figure 2D**).

**Table 3:** Logistic regression analysis of factors influencing NS occurrence.

Variables	Non-NS (N=124)	NS (N=66)	Univariate analysis		Multivariate analysis		
			OR (95CI%)	*p.values*	OR (95CI%)	*p.values*	
CXCL13 (n,%)							
<50pg/mL	69 (55.6%)	6 (9.1%)				
≥50pg/mL	55 (44.4%)	60 (90.9%)	12.55 (5.05-31.20)	<0.001	8.74 (2.95-25.91)	<0.001
CXCL8 (n,%)							
<198.5pg/mL	104 (83.9%)	39 (59.1%)				
≥198.5pg/mL	20 (16.1%)	27 (40.9%)	3.60 (1.81-7.14)	<0.001	3.19 (1.23-8.27)	0.017
CXCL10 (n,%)							
<295pg/mL	21 (16.9%)	20 (30.3%)				
≥295pg/mL	103 (83.1%)	46 (69.7%)	0.47 (0.23-0.95)	0.035	0.26 (0.09-0.73)	0.01
syphilis treated (n,%)							
NO	67 (54%)	43 (65.2%)				
YES	57 (46%)	23 (34.8%)	0.63 (0.34-1.17)	0.141	0.52 (0.22-1.24)	0.141
HIV (n,%)							
NO	26 (21%)	31 (47%)				
YES	98 (79%)	35 (53%)	0.30 (0.16-0.57)	<0.001	0.27 (0.10-0.72)	0.009
Age (n,%)							
<40	68 (54.8%)	27 (40.9%)				
≥40	56 (45.2%)	39 (59.1%)	1.75 (0.96-3.21)	0.069	1.17 (0.50-2.75)	0.716
Sex (n,%)							
female	14 (11.3%)	6 (9.1%)				
male	110 (88.7%)	60 (90.9%)	1.27 (0.47-3.48)	0.639		
Whether there are symptoms or not (n,%)							
NO	111 (89.5%)	30 (45.5%)				
YES	13 (10.5%)	36 (54.5%)	10.25 (4.83-21.73)	<0.001	8.22 (3.07-22.02)	<0.001
LMR (n,%)							
<4.8	104 (83.9%)	53 (80.3%)				
≥4.8	20 (16.1%)	13 (19.7%)	1.28 (0.59-2.76)	0.537		
NLR (n,%)							
<2.5	56 (45.2%)	30 (45.5%)				
≥2.5	68 (54.8%)	36 (54.5%)	0.99 (0.54-1.80)	0.969		
leukocyte (n,%)							
<6.7×10^9^/L	57 (46%)	22 (33.3%)				
≥6.7×10^9^/L	67 (54%)	44 (66.7%)	1.70 (0.91-3.17)	0.094	1.39 (0.44-4.38)	0.574
sero-TRUST (n,%)						
<1:16	65 (52.4%)	22 (33.3%)				
≥1:16	59 (47.6%)	44 (66.7%)	2.20 (1.18-4.10)	0.013	2.68 (1.05-6.81)	0.039
Absolute value of Neutrophil (n,%)							
<3.5×10^9^/L	79 (63.7%)	35 (53%)				
≥3.5×10^9^/L	45 (36.3%)	35 (53%)	1.98 (1.08-3.63)	0.027	0.65 (0.19-2.16)	0.479
Is there a cavity obstruction (n,%)							
NO	115 (92.7%)	61 (92.4%)				
YES	9 (7.3%)	5 (7.6%)	1.05 (0.34-3.26)	0.936		

**Table 4:** Optimized-corrected results.

Indicator	Apparent	Optimism	Corrected	N
C	0.888	0.013	0.875	500
Brier	1.125	-0.012	1.136	500
Intercept	0.000	0.013	-0.013	500
Slope	1.000	0.099	0.901	500

The formula for Model 1 is as follows: Logit (p)=-2.295 **+** 2.402×CXCL13 **+** 1.270 × CXCL10-148 ×CXCL8 **+** 2.252 ×Whether there are symptoms or not -1.534 × HIV co-infection+0.917×sero-TRUST titer. The specific variable assignments are shown in **Supplementary Table 3**.

### 3.5 The correlation between CSF-CXCL13, CSF-CXCL10 and peripheral blood inflammatory factors

The level of CXCL13, CXCL10, and CXCL8 in CSF was shown to have a positive correlation with cerebrospinal fluid white blood cell count (CSF-WBC) and cerebrospinal fluid protein content (CSF-PRO) by Spearman correlation analysis. The level of CSF-CXCL13 had a positive correlation with the absolute value, percentage, and absolute value of peripheral blood neutrophils, and a negative correlation with the percentage of monocytes. The level of CSF-CXCL10 is positively correlated with peripheral blood leukocyte count, absolute lymphocyte count, absolute eosinophil count, eosinophil percentage, and absolute eosinophil count; the level of CSF-CXCL8 is positively correlated with the percentage of peripheral blood mononuclear cells (**Figure 2F**).

### 3.6 Baseline characteristics of prospective follow-up population

In a prospective study, a total of 29 patients had CSF samples and their test results at baseline, 3M, 6M, and 12M. Three patients had incomplete CSF-CXCL13, CSF-CXCL10, and CSF-CXCL8 test results, and two patients could not determine whether treatment response had occurred. Therefore, they were removed and ultimately included in 24 patients. Among the 24 patients, 13 were re examined at 3M, 11 at 6M, and 5 at 12M. 21 patients underwent only one follow-up examination within one year after their initial treatment, while 3 patients underwent two follow-up examinations within one year after treatment. Among the 24 follow-up patients, 11 did not show any treatment response after treatment, while 13 showed treatment response after treatment. Men account for 91.67%, and 50% of patients have neurological related symptoms. As shown in **Table 5**, more than half of the patients who did respond to treatment had baseline serum TRUST titers ≥ 1:16.

**Table 5:** Comparison of baseline characteristics between treatment responsive and non-responsive populations.

Variables	All patients (N=24)	Non treatment response (N=11)	Treatment response (N=13)	*p*.values
Age	39.0 [28.0;58.5]	43.0 [30.5;59.0]	37.0 [28.0;44.0]	0.400
Sexual (n,%)				1.000
Female	2 (8.33%)	1 (9.09%)	1 (7.69%)	
Male	22 (91.67%)	10 (90.9%)	12 (92.3%)	
CXCL13 (pg/mL)	140 [43.0;677]	147 [93.1;321]	256 [40.5;805]	0.839
CXCL10 (pg/mL)	670 [401;1330]	856 [528;1462]	528 [385;930]	0.235
CXCL8 (pg/mL)	126 [95.9;254]	143 [108;244]	97.7 [88.0;146]	0.469
Absolute value of basophil (×10^9^/L)	0.02 [0.01;0.04]	0.02 [0.01;0.04]	0.02 [0.01;0.04]	0.836
Absolute value of eosinophils (×10^9^/L)	0.15 [0.06;0.28]	0.25 [0.09;0.40]	0.10 [0.03;0.26]	0.235
Absolute value of lymphocytes (×10^9^/L)	1.26 [0.98;1.77]	1.19 [0.90;1.34]	1.50 [1.01;2.07]	0.339
Absolute value of monocytes (×10^9^/L)	0.46 [0.31;0.58]	0.46 [0.38;0.58]	0.47 [0.27;0.61]	0.816
Absolute value of Neutrophil (×10^9^/L)	3.14 [2.50;4.30]	3.48 [1.45;4.35]	2.96 [2.61;3.93]	0.794
leukocyte (×10^9^/L)	5.20 [4.40;6.82]	4.79 [3.15;6.73]	5.32 [4.76;6.92]	0.469
NLR	1.94 [1.24;4.00]	1.72 [0.86;4.10]	1.97 [1.30;2.84]	0.622
MRI etiology (n,%)				0.388
No abnormalities	8 (33.33%)	5 (45.5%)	3 (23.1%)	
not checked	10 (41.67%)	4 (36.4%)	6 (46.2%)	
Ischemic foci, cerebral infarction, cerebral atrophy, and cerebral infarction	5 (20.83%)	2 (18.18%)	3 (23.1%)	
meningitis	1 (4.17%)	0 (0.00%)	1 (7.69%)	
syphilis treated (n,%)				0.197
No	14 (58.33%)	8 (72.73%)	6 (46.2%)	
Yes	10 (41.67%)	3 (27.27%)	7 (53.8%)	
sero-TRUST (1:X) (n,%)				0.706
<1:16	11 (45.83%)	6 (54.5%)	5 (38.5%)	
≥1:16	13 (54.87%)	5 (45.5%)	8 (61.5%)	
Chlorine	127 [124;129]	127 [123;130]	127 [126;128]	0.600
CSF-PRO	354 [228;584]	361 [231;529]	301 [219;625]	0.706
Glucose	3.50 [2.85;3.95]	3.50 [2.90;3.75]	3.60 [3.10;3.80]	0.601
Lactate dehydrogenas	17.5 [11.5;25.2]	19.0 [12.0;24.0]	16.0 [11.0;23.0]	0.726
Adenosine deaminase	0.00 [0.00;1.08]	0.00 [0.00;2.50]	0.00 [0.00;0.90]	0.728
Number of nucleated cells	4.00 [1.25;17.0]	5.00 [2.00;7.00]	3.00 [1.00;25.0]	0.661
Memory Loss				1.000
No	8 (72.7%)	4 (66.7%)	4 (80.0%)	
Yes	3 (27.3%)	2 (33.3%)	1 (20.0%)	
talk nonsense:				1.000
No	7 (63.6%)	4 (66.7%)	3 (60.0%)	
Yes	4 (36.4%)	2 (33.3%)	2 (40.0%)	
Unstable walking:				0.455
No	9 (81.8%)	4 (66.7%)	5 (100%)	
Yes	2 (18.2%)	2 (33.3%)	0 (0.00%)	
blurred vision:				1.000
No	9 (81.8%)	5 (83.3%)	4 (80.0%)	
Yes	2 (18.2%)	1 (16.7%)	1 (20.0%)	
Personality change:				0.455
No	10 (90.9%)	6 (100%)	4 (80.0%)	
Yes	1 (9.09%)	0 (0.00%)	1 (20.0%)	

### 3.7 Changes in cytokines in CSF of patients before and after treatment

**Figure 3:**
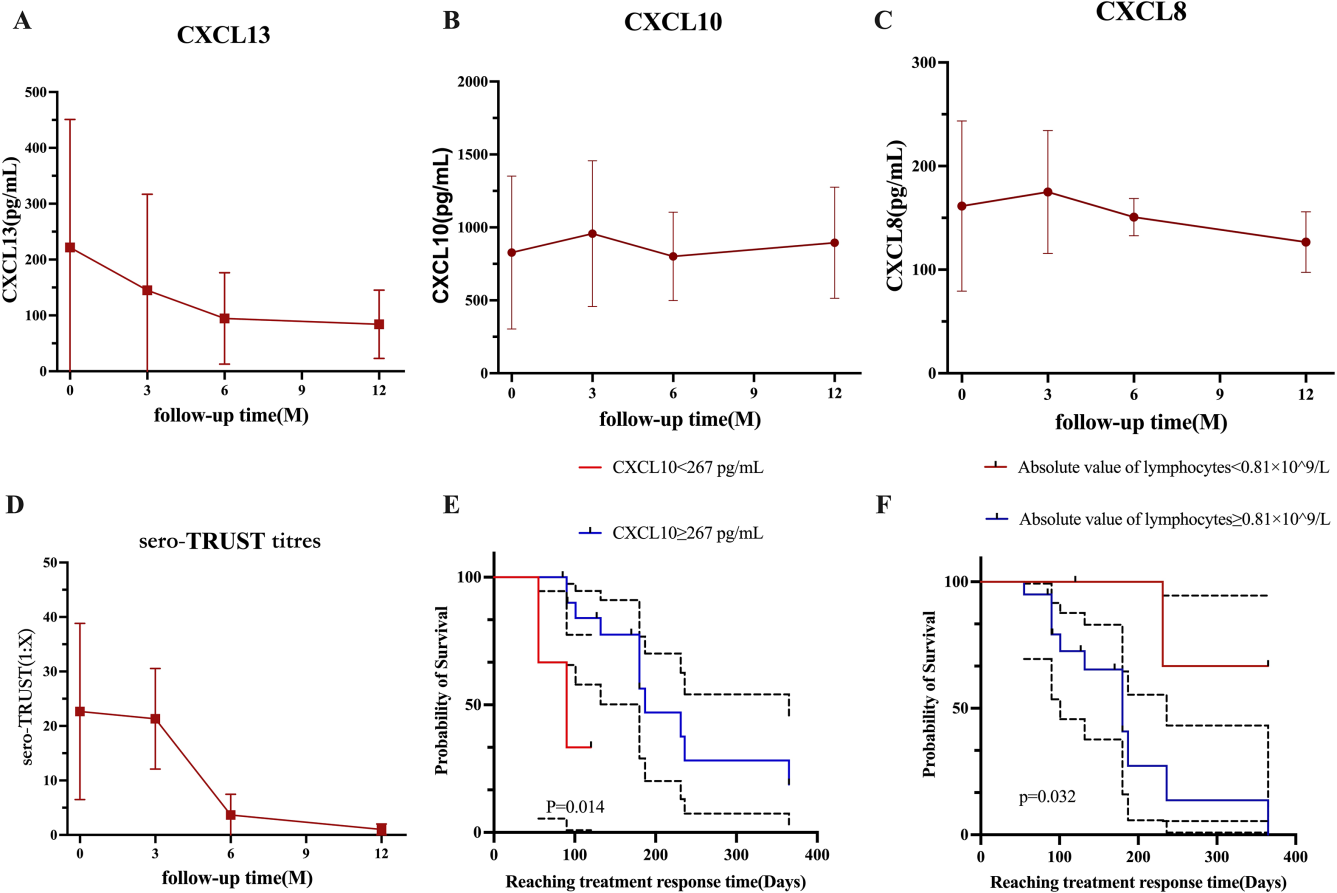
The changes in cytokines during the 12-month follow-up period under different groups. **(A)** Changes in CSF-CXCL13 within 12 months. **(B)** Changes in CSF-CXCL10 over the past 12 months. **(C)** Changes in CSF-CXCL8 over the past 12 months. **(D)** Changes in blood TRUST titers within 12 months. **(E)** The effect of CSF-CXCL10 at different levels on the speed of treatment response. **(F)** The effect of lymphocyte absolute values at different levels on the speed of treatment response.

After treatment (**Figures 3A–D**), the numbers of CSF-CXCL13, CSF-CXCL10, CSF-CXCL8, and sero-TRUST titers decreased, as demonstrated in **Figure 3**. Mann Kendall trend test was used to test that the levels of CSF-CXCL13, CSF-CXCL10, CSF-CXCL, and sero-TRUST titers at 3, 6, and 12 months after treatment did not show a significant downward trend compared to baseline (P>0.05). However, after analyzing the time trend, it was found that CXCL13, CXCL10, CXCL8, and sero-TRUST all had significant stage changes (P<0.001), as shown in **Supplementary Figure 3**.

### 3.8 Factors influencing treatment response

To detect risk factors for treatment response, perform a multivariate analysis of variables with P <0.1 in Cox univariate analysis. CSF-CXCL10 (HR (95% CI): 0.136 (0.022-0.825), P=0.030), CSF-CXCL8 (HR (95% CI): 3.620 (0.864-15.170), P=0.078), and the absolute value of lymphocytes (HR (95% CI): 7.288 (0.901-58.980), P=0.063) were included in the multivariate analysis. The multivariate results showed that CSF-CXCL10 (HR (95% CI): 0.054 (0.007-0.419), P=0.005) reduced the likelihood of response after treatment, while the absolute value of lymphocytes (HR (95% CI): 16.409 (1.567-172.515), P=0.020) increased the likelihood of response after treatment (**Table 6**).

**Table 6:** Analysis of influencing factors for response after treatment - Cox regression analysis.

Variables	All patients (N=24)	Non treatment response (N=11)	Treatment response (N=13)	Univariate analysis			Multivariate analysis	
				HR(95CI%)	*p.values*		HR (95CI%)	*p.values*
CSF-CXCL13(pg/mL)								
<429	15 (62.5%)	8 (72.7%)	7 (53.8%)					
≥429	9 (37.5%)	3 (27.3%)	6 (46.2%)	2.146 (0.707-6.512)	0.178			
CSF-CXCL10(pg/mL)								
<267	3 (12.5%)	1 (9.09%)	2 (15.4%)					
≥267	21 (87.5%)	10 (90.9%)	11 (84.6%)	0.136 (0.022-0.825)	0.030		0.054(0.007-0.419)	0.005
CSF-CXCL8								
<270.46	20 (83.3%)	10 (90.9%)	10 (76.9%)					
≥270.46	4 (16.7%)	1 (9.09%)	3 (23.1%)	3.620 (0.864-15.170)	0.078		1.363(0.887-17.231)	0.072
Absolute value of Neutrophil(n,%)								
<5.11	21 (87.5%)	11 (100%)	10 (76.9%)					
≥5.11	3 (12.5%)	0 (0.00%)	3 (23.1%)	2.374 (0.626-9.007)	0.204			
Absolute value of lymphocytes (n,%)								
<0.81	4 (16.7%)	3 (27.3%)	1 (7.69%)					
≥0.81	20 (83.3%)	8 (72.7%)	12 (92.3%)	7.288 (0.901-58.980)	0.063		16.409(1.567-172.515)	0.020
Absolute value of monocytes (n,%)								
<0.35	7 (29.2%)	2 (18.2%)	5 (38.5%)					
≥0.35	17 (70.8%)	9 (81.8%)	8 (61.5%)	0.475 (0.144-1.565)	0.221			
sero-TRUST(1:X)								
<1:32	14(58.33%)	7 (63.6%)	7 (53.8%)					
≥1:32	10(41.67%)	4 (36.4%)	6 (46.2%)	1.132 (0.374-3.424)	0.826			

### 3.9 The effect of different levels of CSF-CXCL10 and absolute values of peripheral blood lymphocytes on the response speed after treatment

The K-M curve shows that during the initial treatment period of NS, CSF-CXCL10<267 pg/mL may result in a faster treatment response after treatment (**Figure 3E**); If the absolute value of peripheral blood lymphocytes during the initial treatment of NS is ≥ 0.81 × 109/L, the treatment response may be faster after treatment (**Figure 3F**).

## 4 Discussion

CSF-CXCL13 may be have a good ability to differentiate NS from non-NS in the general population, PLWH, and non-PLWH, according to the findings of this study. CSF-CXCL13 has a superior diagnostic advantage in distinguishing NS from other CNS infections, whereas CSF-CXCL10 and CSF-CXCL8 did not perform well in diagnosis. The AUC of the diagnostic model based on CSF-CXCL13 is 0.888. Has good diagnostic ability. According to research, CSF-CXCL13 has an AUC of 0.770 in PLWH, which indicates an increase in specificity and a decrease in sensitivity when compared to the general population.

We have noticed some cytokines related to the occurrence of neurosyphilis, such as CXCL13, IL-6, TNF-α, etc. There is strong evidence for the association between IL-6 and TNF-α and the occurrence of NS. Simin Lu and his team found that IL-6 may be related to the penetration of Treponema pallidum through the blood-brain barrier, while the relationship between CXCL13, CXCL10, CXCL8 and Treponema pallidum penetration through the blood-brain barrier is not clear (9). Although some studies have explored the relationship between CXCL13, CXCL10, and CXCL8 and NS in PLWH and non-PLWH, there are some limitations in the study, such as the relatively narrow definition of neurosyphilis (only relying on CSF-RPR or CSF-VDRL combined with clinical symptoms) and small sample size. The widely used diagnostic criteria for neurosyphilis are comprehensive, requiring compliance with relevant epidemiological history, cerebrospinal fluid TRUST and TPPA positivity; If CSF-TRUST is negative and CSF-TPPA is positive, it may be accompanied by abnormalities and/or neurological symptoms related to CSF-WBC or CSF-PRO and neurosyphilis. This study is based on this comprehensive standard to define neurosyphilis. The original intention of our research is to conduct a more comprehensive verification of cytokines, such as CXCL13. So we include HIV infected people, non-infected people and patients with other central nervous system diseases when collecting samples. We hope to further evaluate the specificity of CXCL13, CXCL10 and CXCL8 in the diagnosis of neurosyphilis, so that NS can be independently diagnosed without interference from other factors. At the same time, we hope to lay a theoretical foundation for the subsequent occurrence mechanism of neurosyphilis. Therefore, in our study, we did not choose IL-6 and TNF-α as positive controls to compare the performance of CXCL13, CXCL10, and CXCL8 as NS. We hope to further prove whether CXCL13 can be used as an independent diagnostic factor for neurosyphilis by improving previous research.

The sensitivity of CSF-CXCL13 in this study was 0.459, which is lower than the 88.9% reported by Ricardo de S Carvalho et al. (10), while the specificity was 0.971, which is similar to the 97.6% result of this study. The diagnostic efficacy of CSF-CXCL13 has been demonstrated by previous studies to undergo dynamic changes at different critical values. In patients with symptomatic neurosyphilis (SNS), its sensitivity and specificity are 41%-90% and 37%-79% respectively (11). In non-PLWH, the AUC of CSF-CXCL13 was 0.852, indicating an increase in sensitivity and specificity compared to the general population, suggesting its higher potential application value in non-PLWH populations. Compared with previously published research results, the sensitivity (0.903) and specificity (0.741) of the non-PLWH population in this study are basically consistent with previous research results (sensitivity 88.89%, specificity 78.95%), with a slight increase in sensitivity (7).

Published studies on the NS mechanism have shown that the CXCL13/CCR5 axis is involved in the recruitment of B cells in the CSF of HIV negative/NS patients (12). It is worth noting that CSF-CXCL13 also plays a similar role in other CNS infections, which may affect its specificity in NS differential diagnosis. However, the results of this study indicate that CSF-CXCL13 has good diagnostic performance (sensitivity 0.813, specificity 0.742), which may be due to the different types of CNS infections included in this study compared to previous studies.

CSF-CXCL13 has a moderate diagnostic efficacy for NS diagnosis, with an AUC of 0.812 (sensitivity: 0.909, specificity: 0.556), as demonstrated by ROC. In contrast, CSF-CXCL10 and CSF-CXCL8 have lower diagnostic sensitivity. The combined diagnostic of CSF-CXCL13, CSF-CXCL10, and CSF-CXCL8 has reached 0.808 with a sensitivity of 0.621 and a specificity of 0.847, but the sensitivity and specificity are still imbalanced. To enhance the diagnostic performance, we utilized logistic regression analysis to create a predictive diagnostic model (Model 1) that is based on CSF-CXCL13 and other indicators. The diagnostic accuracy of this model was significantly improved due to its AUC of 0.888 (sensitivity: 0.864, specificity: 0.815).The sensitivity and specificity of diagnosing NS both reached 0.8. Considering the research sample, we used various methods to avoid overfitting and improve the credibility of the model results during the model construction process. We used 5-fold cross-validation to avoid overfitting during the model construction process. The reason for choosing 5-fold cross-validation to evaluate the model is that it divides the dataset into 5 equal parts and selects 1 part of the data for model training each time, which can effectively avoid overfitting in the training set and lead to poor model performance. There are three commonly used methods for cross-validation: K-fold cross-validation, hierarchical K-fold, and leave one out. The difference between them is that K-fold cross-validation is suitable for data with balanced outcome classification, hierarchical K-fold is suitable for classification problems, and leave one out is suitable for small sample data (dozens of cases). Therefore, our study chose K-fold cross-validation for validation.

During model validation, not only is the bootstrap method used for sampling, but also optimistic corrected estimates are used to correct the results. The calibration results of the model have high reliability, but the size of the sample cannot be ignored, which may lead to a certain degree of bias in the calibration results. It is worth noting that the prediction model based on CSF-CXCL13 is applicable to both HIV positive and negative populations, and its diagnostic efficacy is superior to the AUC value (0.80) reported in previous studies only for HIV negative populations (13). The standard treatment for NS is intravenous infusion of penicillin for 10–14 days. If allergic to penicillin, desensitization treatment or alternative antibiotics may be necessary. Clinical practice has shown that intravenous injection of ceftriaxone sodium for 10–14 days or oral administration of doxycycline for one month can be effective alternative treatment options (14). It is necessary to evaluate the treatment effect through clinical symptom observation and dynamic monitoring of CSF indicators.

Although the mortality rate of NS is relatively low, the clinical cure rate is only 18-44% (15). Due to the limited sample size of the study, the representativeness of existing treatment efficacy data is limited to specific populations. In this study, 24 patients were followed up to observe 12 patients with clinical symptoms and 12 patients without clinical symptoms. Among the 12 patients with clinical symptoms, their clinical symptoms improved after treatment, which is consistent with previous research results (16, 17). All 24 patients completed standardized treatment (penicillin/ceftriaxone sodium) upon discharge, of which 13 achieved serological treatment response after treatment. The research results showed that the proportion of patients with poor prognosis gradually decreased with follow-up time: the proportion of poor prognosis at 3 months, 6 months, and 1 year after treatment was 25%, 12.5%, and 8.33%, respectively. It is worth noting that the treatment response rate of HIV positive patients (56.25%) is slightly higher than that of non-PLWH (50%). This finding is consistent with the research findings of Schnohr et al. They reported that the proportion of patients with poor prognosis at discharge, 1 month, and 3 months was 21%, 19%, and 13%, respectively. The difference in symptom presentation between the two groups of people may be the main reason for the difference in prognosis (18). According to the research findings, treatment response may be influenced by baseline CSF-CXCL10 levels and absolute values of peripheral blood lymphocytes. The higher the baseline CSF-CXCL10 level, the longer it may take to achieve therapeutic response, which may be closely related to the inflammatory state in the patient’s body, but its specific molecular mechanism still needs to be elucidated. Due to the limited number of research samples, this result still needs further validation. As an important immune cell, lymphocytes can reflect the body’s immune status through their absolute peripheral blood levels. An increase in the absolute level of peripheral blood lymphocytes indicates an active immune response in the body, while a low level indicates a weakened immune system function. The treatment response is actually the process of the body clearing Treponema pallidum. Previous studies on the cellular immune status of pregnant syphilis patients have found that patients in the active phase of syphilis typically exhibit immune suppression, while in the recovery phase, cellular immune function can return to normal or near normal levels (19). A higher absolute value of lymphocytes indicates that the patient’s cellular immune function is less inhibited, which may be beneficial for the body to clear Treponema pallidum faster. However, we need to further expand the sample size to verify whether CXCL13, CXCL10, and CXCL8 can serve as indicators of patient efficacy.

We define treatment response as a decrease in CSF-WBC and CSF-PRO levels in CSF to normal levels after treatment, and when chemokines decrease after treatment, we have reason to believe that the treatment is effective. When we conduct long-term follow-up on patients, we can see that this decrease is a long-term trend, but the study sample is small. From the COX regression results, it can be seen that there is a correlation between treatment response speed and baseline CSF-CXCL10 levels after treatment, which may be related to the immune response in the patient’s body. On the other hand, we can determine the closeness of patient follow-up based on the level of CSF-CXCL10 before treatment, which can ensure the effectiveness of treatment and also reduce the medical burden in areas with limited medical resources.

Compared with the other two cytokines, CSF-CXCL13 has better diagnostic ability for NS in any subgroup population. Moreover, CSF-CXCL13 has a good ability to distinguish NS from other CNS infections, while CSF-CXCL10 and CSF-CXCL8 have relatively low specificity in diagnosing NS. Perhaps CSF-CXCL13, CSF-CXCL10, and CSF-CXCL8 are involved to varying degrees in the mechanism of Treponema pallidum penetrating the blood-brain barrier, which may be one of the reasons why CSF-CXCL13 has better diagnostic efficacy while CSF-CXCL10 and CSF-CXCL8 have poorer diagnostic efficacy.

We simultaneously measured the levels of three cytokines in the CSF of patients with different types of CNS infections and found that, except for CXCL13, there was no significant difference in the levels of CXCL10 and CXCL8 between CNS infections and NS (**Figures 1D–F**). The reason for this may be that CXCL13 may be involved in the occurrence of NS, and the mechanism of NS occurrence may be different from cryptococcal meningitis, varicella encephalitis, and other spirochetes that invade the blood-brain barrier. The diagnostic effect of CXCL13 is superior to the other two cytokines, and its specificity is not low. When diagnosed separately, the AUC value is also higher than 0.7. Statistically speaking, the diagnostic effect has reached a moderate level and can be applied independently. In the future, it is necessary to expand the types of CNS infections other than NS and further validate the results. Model 1, based on the combination of cytokines and biomarkers, has a diagnostic ability of over 0.8 and a moderate level of diagnostic performance. When using this model to diagnose NS, the sensitivity (0.864) and specificity (0.815) are further improved. Therefore, we believe that using this model for diagnosing NS in clinical practice is more reliable. In the future, we can also search for other biomarkers closely related to NS and combine them with cytokines such as CXCL13 to further improve diagnostic efficiency. We attempted to use cytokines to differentiate other CNS diseases, such as cryptococcal meningitis and other non-cryptococcal encephalitis; AUC=0.52. The result is not ideal, and the possibility of a small sample size cannot be ruled out. Further validation is needed to determine whether CXCL13, CXCL10, and CXCL8 can be applied to other diseases.

Overall, this study has the following advantages. Firstly, a one-year dynamic follow-up was conducted on syphilis patients after treatment to observe the natural changes in cytokines. Secondly, we combined cytokine and demographic data to further improve the effectiveness of NS cytokine diagnosis, and validated the model using a 5-fold cross-validation calibration curve, DCA), However, this study still has certain limitations. Firstly, patients had poor compliance during the follow-up process, resulting in a small sample size. The research results on prognosis can only serve as a reference and cannot be used as a clear conclusion. In the future, further sample size expansion is needed to observe the relationship between cytokines such as CXCL13 and the treatment response. Secondly, this study is a single center study that lacks sufficient sample size, a diverse population, and internal controls and external validation cohorts to validate the results. Thirdly, the ROC curve only reflects the model’s ability to distinguish between cases and non-cases, which is somewhat unstable. Although we used indicators such as F1-score, accuracy, and recall to observe the results, insufficient sample size remains an unavoidable weakness.

Finally, we would like to say that we conducted a small number of reproducibility experiments in our study. Although the sample size for reproducibility testing was relatively small compared to the total testing sample, this may affect the broad applicability of our research results, resulting in our findings only being applicable to research centers with similar population characteristics to ours. However, despite this, the repeatability test results of the samples indirectly reflect the acceptability of our research data.

The research results indicate that CXCL13 exhibits good efficacy in distinguishing NS from other CNS infections, which may be due to differences in the pathways of Treponema pallidum and other pathogens in disrupting the blood-brain barrier. Future research should focus on exploring the specific mechanisms by which different pathogens disrupt the blood-brain barrier, providing a deeper theoretical basis for the diagnosis and treatment of NS. At the same time, expand the sample size and actively seek external datasets to validate the model, in order to apply it to clinical practice and provide broader possibilities.

## 5 Conclusion

CSF-CXCL13 has a high diagnostic performance for distinguishing NS (NS and Non-NS; NS and CNS infectious), but CSF-CXCL10 and CSF-CXCL8 have a low discriminative ability. The patient’s CSF levels of CSF-CXCL13, CSF-CXCL8, and CSF-CXCL10 decreased after receiving NS treatment. Model 1 constructed based on CSF-CXCL13 can effectively balance the sensitivity and specificity of diagnosing NS. According to Cox regression analysis, the absolute values of CSF-CXCL10 and lymphocytes prior to treatment may be a reliable indicator of patient treatment response. The Kaplan Meier survival curve shows that a faster treatment response is likely due to lower CSF-CXCL10 levels and higher lymphocyte absolute values.

## Data Availability

The raw data supporting the conclusions of this article will be made available by the authors, without undue reservation.

## Supplementary material

Supplementary Figure 1 Research flowchart.

Supplementary Figure 2 Standard curve. (A) CXCL13 standard curve. (B) CXCL8 standard curve. (C) CXCL10 standard curve.

Supplementary Figure 3 Time series analysis within 1 year (A) CXCL13 time series analysis. (B) CXCL10 time series analysis. (B) CXCL8 time series analysis. (B) Sero TRUST time series analysis.
