# Supplementary figures and images for: The diagnostic and prognostic value of CXCL13, CXCL10, and CXCL8 in patients with neurosyphilis
Source: Front Immunol. 2025 Oct 27;16:1654251. doi: 10.3389/fimmu.2025.1654251 (PMC12597757; doi:10.3389/fimmu.2025.1654251)

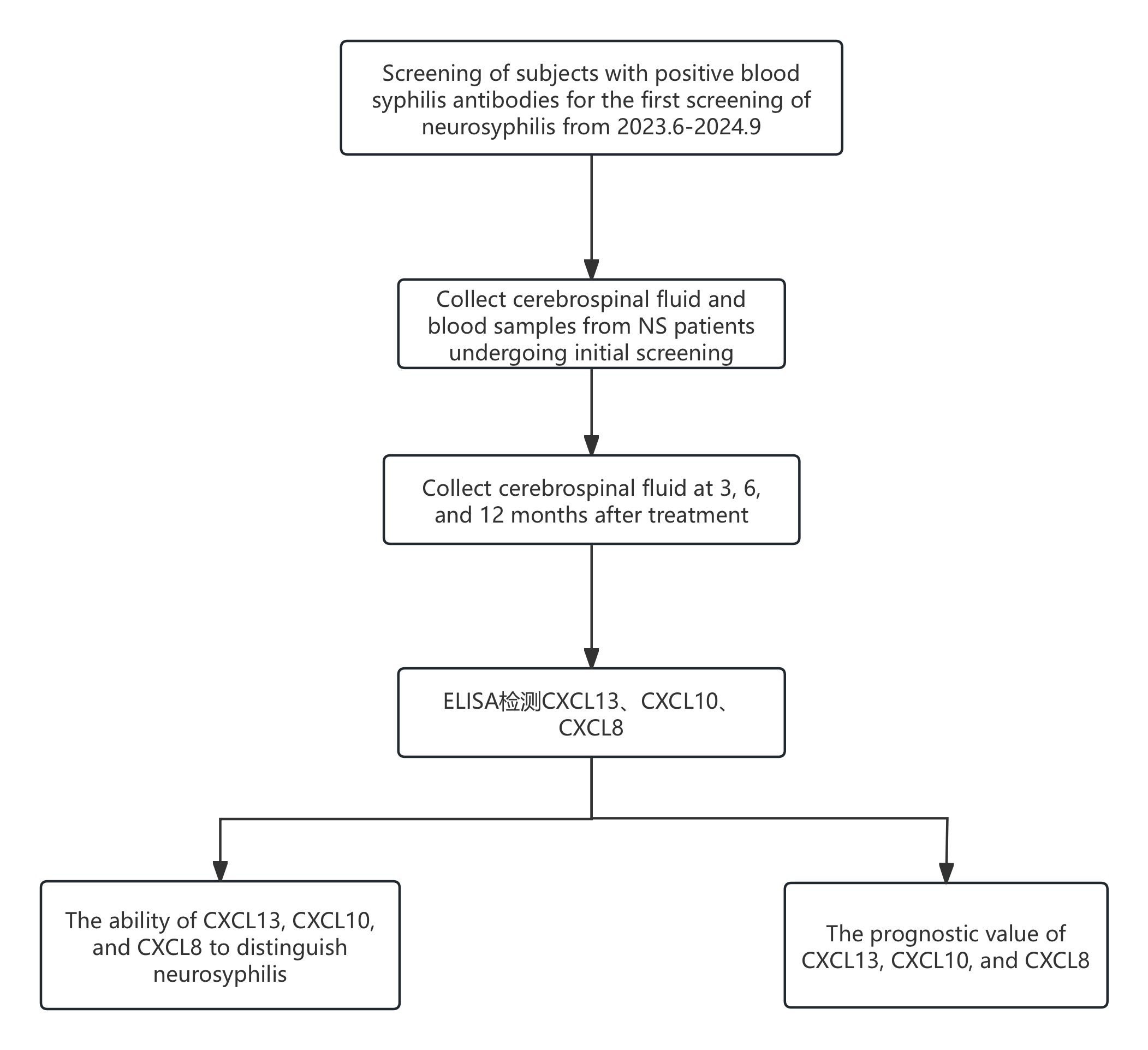

Supplement: Supplementary Figure 1 — Research flowchart. [file Image1.jpeg]

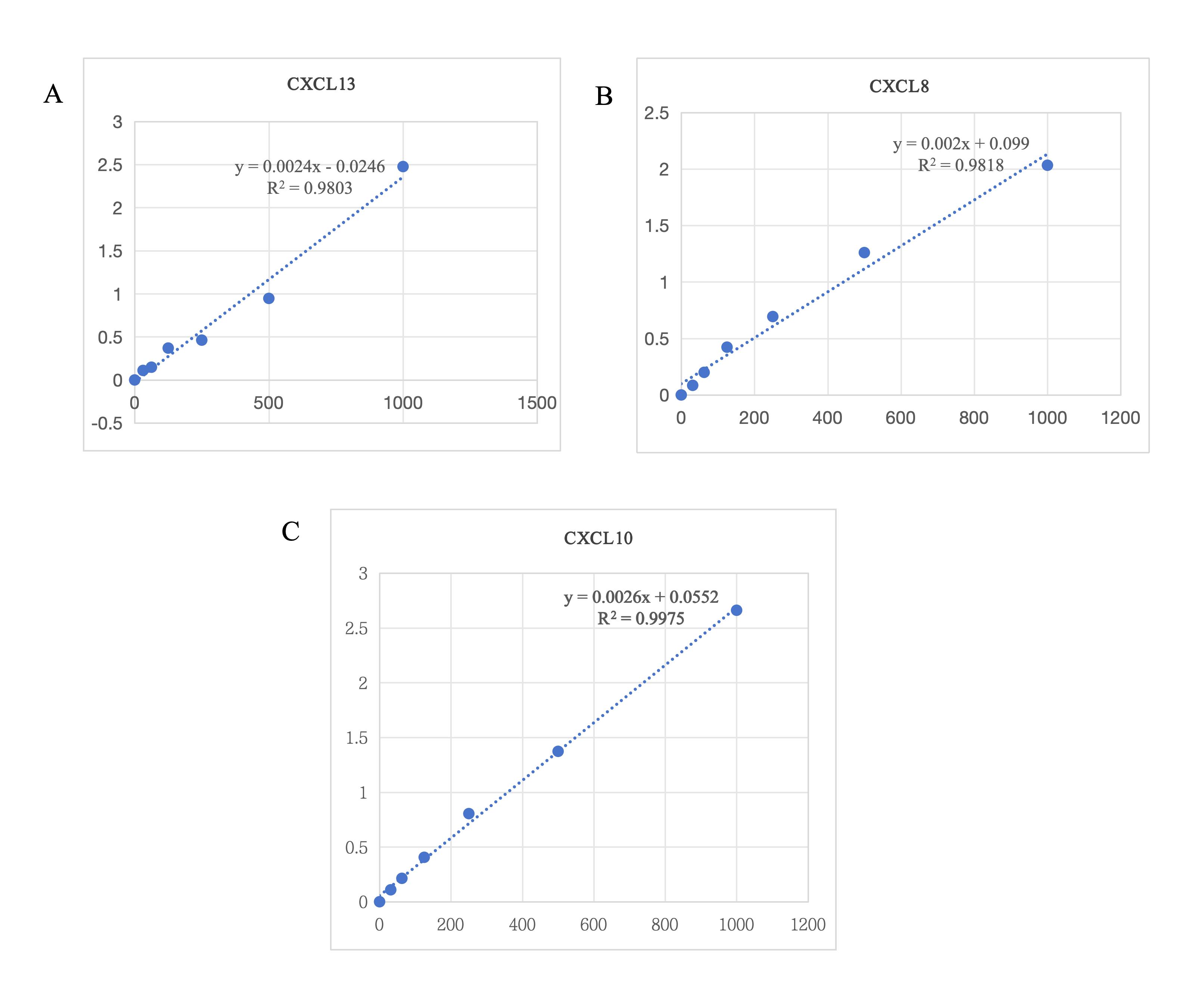

Supplement: Supplementary Figure 2 — Standard curve. (A) CXCL13 standard curve. (B) CXCL8 standard curve. (C) CXCL10 standard curve. [file Image2.jpeg]

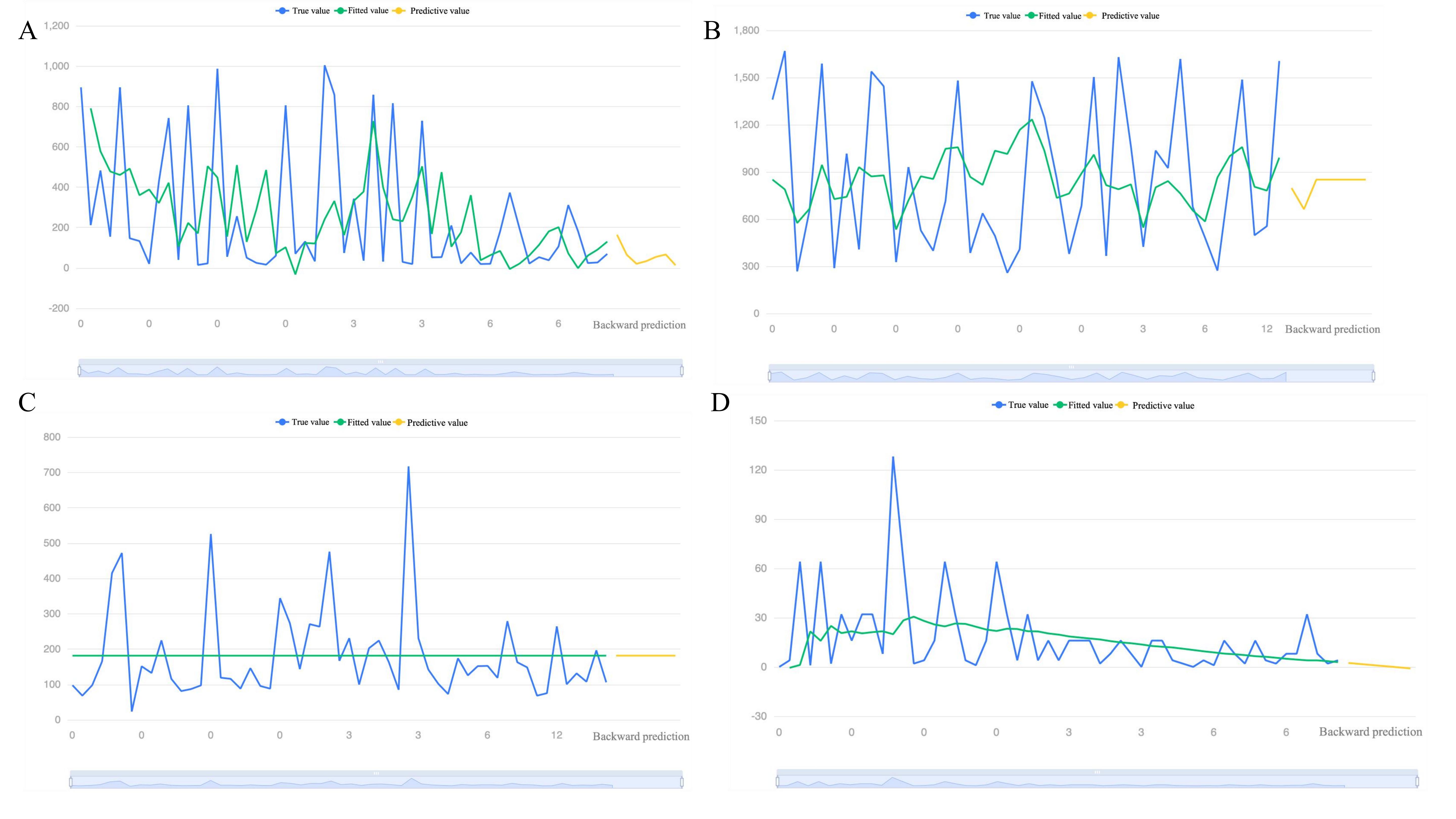

Supplement: Supplementary Figure 3 — Time series analysis within 1 year (A) CXCL13 time series analysis. (B) CXCL10 time series analysis. (B) CXCL8 time series analysis. (B) Sero TRUST time series analysis. [file Image3.jpeg]
