# Supplementary material for: The diagnostic and prognostic value of CXCL13, CXCL10, and CXCL8 in patients with neurosyphilis
Source: Front Immunol. 2025 Oct 27;16:1654251. doi: 10.3389/fimmu.2025.1654251 (PMC12597757; doi:10.3389/fimmu.2025.1654251)
Supplement: Supplementary file 4 [file Table1.docx]

Supplymentary material Table 1 Repetitive testing of different cytokines

| Mean | standard deviation | coefficient of variation |
| --- | --- | --- |
| CXCL8 |  |  |
| 0.96 | 0.25 | 26% |
| 1.03 | 0.25 | 24% |
| 0.40 | 0.06 | 16% |
| 0.44 | 0.03 | 7% |
| 0.50 | 0.17 | 35% |
| 1.21 | 0.25 | 21% |
| 0.28 | 0.02 | 6% |
| 0.38 | 0.03 | 7% |
| CXCL10 |  |  |
| 0.14 | 0.00 | 1% |
| 2.93 | 1.33 | 46% |
| 3.51 | 0.66 | 19% |
| CXCL13 |  |  |
| 0.65 | 0.18 | 27% |
| 2.35 | 0.64 | 27% |
| 0.54 | 0.04 | 7% |
| 0.67 | 0.17 | 26% |
