# Supplementary material for: The diagnostic and prognostic value of CXCL13, CXCL10, and CXCL8 in patients with neurosyphilis
Source: Front Immunol. 2025 Oct 27;16:1654251. doi: 10.3389/fimmu.2025.1654251 (PMC12597757; doi:10.3389/fimmu.2025.1654251)
Supplement: Supplementary file 5 [file Table2.docx]

Supplymentary material :Table2 Model1 variable collinearity

| Variable | VIF | Tolerance |
| --- | --- | --- |
| CXCL13 | 1.070923 | 0.9337741 |
| CXCL10 | 1.145808 | 0.8727464 |
| CXCL8 | 1.166102 | 0.8575577 |
| sero-TRUST | 1.210814 | 0.8258909 |
| Whether there are symptoms or not | 1.13299 | 0.8826207 |
| HIV co-infectious | 1.128393 | 0.8862158 |
