# Supplementary material for: The diagnostic and prognostic value of CXCL13, CXCL10, and CXCL8 in patients with neurosyphilis
Source: Front Immunol. 2025 Oct 27;16:1654251. doi: 10.3389/fimmu.2025.1654251 (PMC12597757; doi:10.3389/fimmu.2025.1654251)
Supplement: Supplementary file 6 [file Table3.docx]

Supplementary material :Table 3 Variable Assignment Table of MODEL1

| variable | quantizer |
| --- | --- |
| CSF-CXCL13 | CSF-CXCL13<50pg/mL：0，CSF-CXCL13≥50pg/mL：1 |
| CSF-CXCL10 | CSF-CXCL10<198.5pg/mL：0，CSF-CXCL10≥198.5pg/mL：1 |
| CSF-CXCL8 | CSF-CXCL8<295pg/mL：0,CSF-CXCL8≥295pg/mL：1 |
| sero-TRUST titer | sero-TRUST titre<1:16 : 0，sero-TRUSTtitre≥1:16 : 1 |
| Are there any symptoms related to the nervous system | Symptoms:0，Asymptoms:1 |
| HIV co-infection | non-HIV:0，HIV:1 |
